# Persistent Smell Disorders After COVID-19 Infection and Their Impact on Quality of Life

**DOI:** 10.7759/cureus.58469

**Published:** 2024-04-17

**Authors:** Yahya A Fageeh, Ahmad S Altuwaireqi, Abdulaziz B Alghuraibi, Muath S Alotaibi, Lara E Alsulimany, Eman A Altooarki

**Affiliations:** 1 Otorhinolaryngology, Surgery Department, College of Medicine, Taif University, Taif, SAU; 2 College of Medicine, Taif University, Taif, SAU; 3 General Practice, Emergency Department, Alhada Armed Forces Hospital, Taif, SAU

**Keywords:** emotional distress, pcr-confirmed, cross-sectional, lingering consequences, parosmia, anosmia, hyposmia, quality of life, smell dysfunction, olfactory disorders

## Abstract

Background

The COVID-19 pandemic has led to various health challenges, including the disruption of people’s sense of smell. Olfactory disorders have been reported as a lingering consequence of COVID-19, with diverse patterns of smell dysfunction experienced by patients.

Objectives

This study aimed to investigate the impact of persistent smell disorders on the quality of life of individuals who recovered from COVID-19 in Taif, Saudi Arabia.

Methodology

A descriptive cross-sectional study was conducted in Taif, Saudi Arabia, between October 2023 and January 2024. The study included adults with a history of PCR-confirmed COVID-19 infection in Taif city. Data were collected using a validated online survey employing a convenience sampling technique. Statistical analysis was carried out using IBM SPSS Statistics for Windows, Version 26.0 (Released 2019; IBM Corp., Armonk, New York, United States), and chi-squared tests were used to assess the relationships.

Results

The study included 429 participants. A total of 52.7% of the respondents reported a loss of smell after recovering from COVID-19, and 14.9% reported a persistent loss of their sense of smell. The most common types of smell disorders experienced were hyposmia, anosmia, and parosmia. The study revealed emotional distress, changes in eating habits, and social impact among participants with smell disorders.

Conclusion

This study highlights the high prevalence of persistent smell disorders among individuals who recovered from COVID-19 in Taif, Saudi Arabia. The findings emphasize the complex nature of these disorders and their impact on patients’ quality of life. This study contributes valuable information that can inform healthcare practices and support services for individuals experiencing post-COVID-19 smell disorders.

## Introduction

The emergence of the COVID-19 pandemic has brought several health challenges, with a growing body of evidence highlighting its diverse and lingering effects on various physiological systems. One of the enduring consequences frequently mentioned by individuals who have recovered from COVID-19 is the disruption of their sense of smell [1]. Many studies have highlighted the significance of olfactory disorders as a lingering consequence of COVID-19 infection. However, different studies across the globe have reported diverse patterns of post-COVID-19 smell disorders, including a reduced sense of smell (hyposmia), complete loss of smell (anosmia), distorted smell (parosmia), unpleasant smell (cacosmia), or smelling an odor that is not present (phantosmia) [2,3].

Although the exact mechanisms underlying these enduring smell disorders are still being investigated, various factors are being considered as potential contributors. Severe acute respiratory syndrome coronavirus 2 (SARS-CoV-2) virus has established a propensity for neurotropism, indicating its ability to infect and damage sustentacular cells that provide support to olfactory sensory neurons, which directly influence the sense of smell. The regeneration rate of sustentacular cells and subsequent restoration of olfaction can vary among individuals. Moreover, the body's immune response to the virus may result in inflammation in the nasal and olfactory regions. This inflammatory response can further damage olfactory neurons and disrupt the transmission of smell signals to the brain, resulting in longstanding olfactory dysfunction [4,5].

Many studies have been conducted to determine whether a relationship exists between olfactory dysfunction and reduced quality of life. It is difficult to imagine how life can be difficult and unbearable after recovering from a life-threatening disease such as COVID-19. People with smell disorders often experience changes in lifestyle, altered eating habits, and social withdrawal [6].

The smell is essential to enjoying experiences such as cooking, eating, and nature. Individuals with post-COVID-19 persistent smell disorders often feel deprived of this privilege [7]. After recovery from COVID-19, eating a balanced diet is essential; loss of a sense of smell could result in reduced appetite, weight loss, or changes in food choices, affecting a patient's health. A sense of smell is pivotal in social interactions. Hence, persistent olfactory disturbances may contribute to social withdrawal or avoidance due to concerns about personal hygiene and low self-esteem. This consequently leads to psychological effects such as increased stress, frustration, and even symptoms of depression or anxiety. The persistent nature of the condition may contribute to a sense of loss and frustration [8].

In Saudi Arabia, it has been observed that smell disorders are relatively common after COVID-19 infection, and in some cases, the symptoms may persist even after recovery [9]. Hence, it is imperative to understand the impact of persistent smell disorders on the quality of life of COVID-19 patients in Taif city. This research aims to address this gap in the literature by providing valuable information that can help healthcare professionals tailor support services and improve the overall well-being of individuals affected by persistent smell disorders after COVID-19. This study sought to determine the prevalence and types of smell disorders experienced by patients, explore the duration and potential long-term consequences of these disorders, and assess the emotional and social impact of smell disorders on patients' daily lives and well-being.

## Materials and methods

Study design and setting

This descriptive cross-sectional study was conducted in Taif, Saudi Arabia, between October 2023 and January 2024. This study aimed to determine the effect of persistent olfactory disorders on the quality of life of individuals who recovered from COVID-19.

Study population

The study population included adults with a history of PCR-confirmed COVID-19 infection before one year within the boundaries of Taif city. We excluded individuals under 18 years of age or with preexisting smell disorders prior to COVID-19 infection. We also excluded respondents who met the inclusion criteria but were unwilling to participate in the study.

Sample size

We adopted the Cochrane sample size formula, given by the g formula of n = Z^2^(1 − p)/d^2^, where n is the sample size and Z is the critical statistic for the 95% confidence interval. An anticipated proportion of 50% was used. We used a margin of error (d) of 0.05. After the computation, a sample size of 384 was obtained. However, we chose to increase the sample size to 429 respondents.

Data collection

A validated online survey (Microsoft form) was administered to the target population via social media such as WhatsApp and Telegram. Prior to administering the questionnaire, we conducted a pilot study on a small sample of participants to ensure the clarity and understandability of the questions. The participants involved in the pretest were not involved in the final survey. The questionnaire consisted of three main sections: the first section collected social-demographic information, the second section collected the clinical characteristics of the respondents, and the third section assessed the quality of life and attitudes of participants toward changes in smell obtained from the questionnaire of olfactory disorder negative statements (QOD-NS) [10].

Statistical analysis

The data were collected and entered into Excel spreadsheets for cleaning. After cleaning, the data were coded for statistical analysis. IBM SPSS Statistics for Windows, Version 26.0 (Released 2019; IBM Corp., Armonk, New York, United States) was used for all data analysis. Categorical variables are presented as counts and percentages. The chi-squared test was used to determine associations between categorical variables. Statistical significance was defined as a P-value of 0.05 or less.

Ethical considerations

This study obtained ethical approval from Taif University's Ethics Committee (reference number 44-386). Confidentiality and informed consent were some of the important ethical considerations. Throughout the study, the respondents were explicitly assured of the strict confidentiality of their information. Additionally, they were informed that they had the right to revoke consent at any juncture, emphasizing their autonomy and the ability to cease participation at their discretion.

## Results

The study included 429 participants from Taif city. The results revealed that the majority of the respondents were females (59.2%; n=254), while the rest were males (40.8%; n=175). In terms of age distribution, the majority (68.5%; n=294) were aged between 18 and 29 years (Table 1).

**Table 1:** Sociodemographic distribution of the participants (n=429).

Variables	Category	Frequency	Percentage
Gender	Male	175	40.8
	Female	254	59.2
Age	18-29	294	68.5
	30-39	48	11.2
	40-49	45	10.5
	50-59	32	7.5
	60 and above	10	2.3
Nationality	Saudi	406	94.6
	Non-Saudi	23	5.4

Notably, the majority of the respondents experienced a change in their sense of smell during active COVID-19 infection (82.5%; n=354), and 52.7% (226) experienced smell disturbance even after recovery. When we assessed the type of smell disorder, 153 (35.7%) respondents reported hyposmia, followed by anosmia (17.5%; n=75) and parosmia (10.3%; n=44). A significant number of individuals experienced some form of smell disorder after recovering from COVID-19, with the majority, 159 (37.1%), experiencing the problem only within one week after recovery. In comparison, 64 (14.9%) participants had persistent smell disorders for more than a year (Table 2).

**Table 2:** Characteristics of smell disorders related to COVID-19 among the participants (n=429).

Variable	Category	Frequency	Percentage
Smell disorder during active infection	Yes	354	82.5
	No	75	17.5
Smell disorder after recovery	Yes	226	52.7
	No	203	47.3
Type of smell disorder	Anosmia	75	17.5
	Hyposmia	153	35.7
	Cacosmia	36	8.4
	Phantosmia	10	2.3
	Parosmia	44	10.3
Duration of smell disorder	Less than a week	159	37.1
	One month	92	21.4
	Three Months	26	6.1
	Six Months	18	4.2
	Year	8	1.9
	More than year	64	14.9

Many respondents expressed concern about their ability to adapt to changes in their sense of smell and its impact on their daily activities, particularly during meals. Approximately 35.2% of the participants (n=151) expressed concern about getting used to changes in their sense of smell. Additionally, 28% of the respondents (n=120) said they feared exposure to dangers such as gas or rotten food due to difficulties with smelling. Interestingly, changes in smell had an impact on the social and relationship aspects of the respondents. Approximately 20% (n=86) of the participants fully agreed that changes in smell led to the avoidance of social groups. The results further indicate that changes in smell also affected enjoyment and emotional response. Approximately 30% (n=130) of the respondents claimed that they reduced their enjoyment of food and drinks due to changes in smell (Table 3).

**Table 3:** Quality of life and attitudes of participants post-COVID-19 with persistent smell disorder (n=429). The data are presented as frequencies (N) and proportions (%)

Statements	Response of participants, N (%)			
	Agree	Agree partly	Disagree partly	Disagree
I am really worried that I will never adapt to the changes in my sense of smell	151 (35.2)	106 (24.7)	98 (22.8)	74 (17.2)
The changes in my sense of smell annoy me particularly during meals	150 (35)	116 (27)	91 (21.2)	72 (16.8)
Due to the shifts in my sense of smell, my eating habits have changed	115 (26.8)	96 (22.4)	125 (29.1)	93 (21.7)
Because of the changes in my sense of smell, I try harder to relax	108 (25.2)	97 (22.6)	126 (29.4)	98 (22.8)
Changes in sense of smell instill fear in me, particularly concerning potential exposure to specific hazards such as gas or spoiled food	120 (28)	76 (17.7)	127(29.6)	106 (24.7)
I am always aware of the changes in my sense of smell	151 (35.2)	105 (24.5)	82 (19.1)	91 (21.2)
I avoid social gatherings due to the alterations in my sense of smell	86 (20)	78 (18.2)	151 (35.4)	113 (26.3)
The changes in my sense of smell make me feel isolated	78 (18.2)	75 (17.5)	146 (34.0)	130 (30.3)
The changes in my sense of smell have impacted my relationship with my spouse or partner	74 (17.2)	63 (14.7)	169 (39.4)	123 (28.7)
Due to changes in my sense of smell, I find less enjoyment in beverages and food compared to my previous experiences	130 (30.3)	107 (24.9)	107 (24.9)	85 (19.8)
The changes in my sense of smell make me feel angry	119 (27.7)	93 (21.4)	114 (26.6)	104 (24.2)
The changes in my sense of smell create challenges for my participation in everyday activities	81 (18.9)	91 (21.2)	131 (30.5)	126 (29.4)
The changes in my sense of smell cause most of my problems	82 (19.1)	73 (17)	146 (34)	128 (29.8)
Because of the changes in my sense of smell, I go to restaurants less often than I used to	88 (20.5)	104 (24.2)	133 (31)	104 (24.2)
Because of the changes in my sense of smell, I have weight problems	81 (18.9)	85 (19.8)	148 (34.5)	115 (26.8)
I have reduced my visits to friends, relatives, or neighbors due to the changes in my sense of smell	71 (16.6)	81 (18.9)	152 (35.4)	125 (29.1)
Because of the changes in my sense of smell, I feel more anxious than I used to feel	89 (20.7)	98 (22.8)	133 (31)	109 (25.4)

Although the prevalence of persistent smell disorders appeared to be highest in individuals aged 30-39 years and lowest in those aged over 60 years, these differences were not statistically significant. Furthermore, the study revealed no significant difference in the likelihood of persistent smell disorders between men and women or between Saudi and non-Saudi participants (Table 4).

**Table 4:** Relationships between persistent smell disorders and demographic characteristics of participants (n=429). The data are presented as frequencies (N) and proportions (%); the P-value was considered significant at the P<0.05 level; χ^2^: chi-squared test.

Variables	Category	Total	Persistent smell disorders		χ^2^	P-value
			Yes N (%)	No N (%)		
Gender	Male	175	84 (48)	91 (52)	2.5977	0.1070
	Female	254	142 (55.9)	112 (44.1)		
Age	18-29	294	150 (51)	144 (49)	3.2626	0.5149
	30-39	48	29 (60.4)	19 (39.6)		
	40-49	45	25 (55.6)	20 (44.4)		
	50-59	32	15 (46.9)	17 (53.1)		
	60 and above	10	7 (70)	3 (30)		
Nationality	Saudi	406	212 (52.2)	194 (47.8)	0.6538	0.4188
	Non-Saudi	23	14 (60.9)	9 (39.1)		

The study further investigates the prevalence of persistent smell disorders among participants with different types of smell disorders. Of the total participants, 318 were able to identify their specific type of smell disorder, while the rest were uncertain. Among those who reported their type of smell disorder, we found that anosmia had the lowest prevalence of persistent smell disorders (64%; n=64), while cacosmia had the highest prevalence (86.1%; n=31). The prevalence of persistent smell disorders, such as hyposmia, parosmia, and phantosmia, fell somewhere in between. However, this variance in incidence was not deemed statistically significant (Table 5).

**Table 5:** The distribution of patients with persistent smell disorders by type of smell disorder (n=318). The data are presented as frequencies (N) and proportions (%); the P-value was considered significant at the P<0.05 level; χ^2^: chi-squared test.

Type of smell disorder	Persistent smell disorders		χ^2^	P-value
	Yes N (%)	Yes N (%)		
Anosmia	48 (64)	27 (36)	6.7529	0.1495
Hyposmia	103 (67.3)	50 (32.7)		
Cacosmia	31 (86.1)	5 (13.9)		
Phantosmia	7 (70)	3 (30)		
Parosmia	33 (75)	11 (25)		

## Discussion

The COVID-19 pandemic has led to post-COVID-19 smell disorders, which are common enduring complications of COVID-19 infection [11]. Our study aimed to investigate the impact of post-COVID-19 smell disorders on patients' quality of life.

The key finding of this study was that more than half of the respondents (52.7%) reported experiencing a loss of smell after recovering from COVID-19. This prevalence is higher than that reported in previous research conducted in Saudi Arabia, which indicated a prevalence of 43.3% [12].

This study sought to identify the different types of smell disorders experienced by COVID-19 patients. The results showed that the most common smell disorders after COVID-19 infection were hyposmia, anosmia, and parosmia. They also showed that a higher percentage of patients with cacosmia reported having persistent smell disorders, which was not statistically significant. This finding is consistent with previous studies, which also found different types of smell disorders in COVID-19 patients but did not indicate any particular type having a heightened incidence of persistence [10, 11].

Smell and taste disorders can persist in up to 11% of patients. If the smell disorder persists for more than a year, it is unlikely that the patient will recover their sense of smell [11]. The study also sheds light on the variable duration of these disorders, with some resolving within a week while others persisting longer. This highlights the potential for long-term consequences of COVID-19 and the need for further exploration of these lingering sensory issues. Existing research suggests a trend toward improvement within the first month, although a significant number of patients may experience a prolonged loss [13].

The SARS-CoV-2 virus causes COVID-19-related loss of smell, but the exact mechanism is complex. The virus can damage sustentacular cells that support olfactory sensory neurons, leading to smell impairment. The recovery timeline for this damage varies from person to person. Additionally, virus-triggered inflammation can also directly harm olfactory neurons, resulting in longer-lasting smell dysfunction [14,15].

The impact of these changes on patients' well-being and daily lives is multifaceted. Many participants expressed emotional distress and concerns about adapting to their altered sense of smell. Furthermore, a substantial portion reported changes in eating habits and social interactions, with some avoiding social gatherings altogether. These findings align with previous studies documenting a link between post-COVID-19 smell disorders and emotional hardship, behavioral modifications, and social challenges [16,17].

Smell disorders lingering after COVID-19 infection significantly impact patients' quality of life, as measured by the well-validated QOD-NS questionnaire [10]. This negative impact appears to be more severe than in patients with smell loss from other causes. The reasons for long-term olfactory recovery in COVID-19 remain unclear, but the sudden onset and lack of predictive knowledge may contribute to a heightened psychological burden in these patients. Moreover, the longer the smell disorders persist, the worse the impact on quality of life, highlighting a clear positive correlation between symptom duration and quality of life score [18,19].

Although several studies have investigated smell disorders associated with COVID-19, the risk factors for these disorders remain unclear. Some studies suggest risk factors such as age, sex, underlying chronic diseases, associated symptoms, severity of illness, or virus variants [11, 18].

The study involved a diverse group of 429 participants, which makes the findings more significant and applicable to a broader population. Using a well-established quality-of-life questionnaire ensured that the data collection was efficient and reliable. However, the study also has some limitations. A cross-sectional design can establish relationships between factors but cannot ascertain causation. Furthermore, relying on self-reported data introduces potential recall bias and subjective interpretation. Last, using only a questionnaire might oversimplify the complex nature of persistent smell disorders.

## Conclusions

Approximately 52.7% of participants experienced a smell disturbance after recovering from COVID-19, with various types of smell disorders observed. The duration of these disorders varied widely, emphasizing their transient nature in some cases and revealing that 14.9% of patients experienced enduring effects. This research underscores the importance of understanding the long-term consequences of COVID-19 and the potential persistence of sensory symptoms. The study revealed that patients with persistent smell disorders often experienced emotional distress, behavioral changes, and social challenges, with concerns about adaptation and impacts on daily activities and relationships. Hence, it is imperative to provide psychological support through psychotherapy or counseling to assist patients in coping with emotional distress and adapting to changes in behavior.
